# Traditional Chinese Medicine in Neuroprotection after Brain Insults with Special Reference to Radioprotection

**DOI:** 10.1155/2018/2767208

**Published:** 2018-11-26

**Authors:** Xiao Chun Peng, Jiang Rong Huang, Si Wei Wang, Lian Liu, Zhen Zhen Liu, Gautam Sethi, Bo Xu Ren, Feng Ru Tang

**Affiliations:** ^1^Medical School of Yangtze University, Jingzhou 434000, China; ^2^Department of Pharmacology, Yong Loo Lin School of Medicine, National University of Singapore, Singapore 117600; ^3^Radiation Physiology Laboratory, Singapore Nuclear Research and Safety Initiative, National University of Singapore, 1 CREATE Way #04-01, CREATE Tower, Singapore 138602

## Abstract

With rapidly increased construction of nuclear power plants worldwide to reduce energy shortage and subsequent environment contamination, routine use of radiotherapy and radiodiagnosis equipment in the clinical medicine, the research on the health effect of radiation exposure has become a very important area to explore. Traditional Chinese Medicine (TCM) may be an ideal candidate therapy as it usually produces fewer side effects even with long-term administration. In this paper, we reviewed current therapeutic approaches to prevent radiation-induced brain neuropathological and functional changes. Neuroprotective effects of TCM in different brain injury models have been briefly summarized. We then reviewed the neuroprotective and radioprotective effect of TCM in different radiation exposure models and discussed the potential molecular mechanism(s) of the neuroprotective and radioprotective effect of TCM. The conclusions and future research directions were made in the last part of the paper.

## 1. Introduction

*Radiation-Induced Brain Dysfunction*. Nuclear accidents such as radiation leakage from the Fukushima nuclear power plant in Japan in 2011, extensive use of X-ray, Computed Tomography (CT scan), Positron Emission Tomography (PET) in medical diagnosis, radiotherapy (RT) for treatment of human cancers, space travel, and atomic weapons testing have significantly increased the chance of radiation exposures [1]. Exposure to high doses/dose rates of radiation leads to an increased risk for cancer and noncancerous diseases including atherosclerotic, cardiovascular, cerebrovascular, and neurodegenerative effects [2]. Irradiation of eukaryotic cells induces damage to proteins, lipids, and DNA directly or indirectly due to free radical formation. Cell signaling events in response to radiation depend on environmental conditions besides genetic and physiological features of the biological systems [3]. In the mammalian brain, severe structural and functional injury occurs after acute or fractionated high dose radiation exposure [4]. Low doses/dose rates of radiation exposure may produce cognitive impairment even without any significant morphological alterations [5]. Ionizing radiation (IR) provokes cognitive deficits, especially during childhood and adolescence.

Different psychiatric disorders, including depression, bipolar disorder, and schizophrenia, may be related to hippocampal neurogenesis disturbances. There is evidence of an increased incidence of schizophrenia spectrum disorders following exposure to atomic bombing radiation, radiotherapy, or environment with high natural IR level [6]. Alzheimer's disease (AD) is a human neurodegenerative disease, and its global prevalence has been predicted to increase dramatically in the following decades. Mounting evidence suggests that exposure to IR may result in the development of AD [7]. On the other hand, retrospective studies involving the general population and those with brain radiotherapy did not show any association between RT and Alzheimer's disease [8, 9]. From a therapeutic point of view, so far, only Amifostine has been used as an important* adjunct* to radiotherapy to reduce radiation-induced damage to normal tissues or cells, particularly in skin, intestine, marrow, mucosa, and salivary glands with lesser activity in kidney and lung and none in brain [10]. However, toxic side effects of Amifostine have restricted its use in clinical treatment of radiation-induced diseases [11]. Therefore, it is important to develop compounds which can protect against radiation-induced brain damage with less side-effect.

In the behavioral tests to evaluate the effect of Traditional Chinese Medicine (TCM) on learning and memory, Morris water maze test has been commonly used. The maze consists of a pool, with a hidden platform submerged just below the water surface. During the Morris water maze test, the rat or mouse learns to escape from the water by locating a hidden platform with the help of visual cues. The learning ability is quantified as escape time. The shorter escape time a mouse or a rat needed to find the central platform, the better its spatial memory [12].

In this review paper, a comprehensive literature research was carried out using key words “Traditional Chinese Medicine (TCM), radiation or irradiation, neuronal damage, brain, neuroprotection, or radioprotection” by means of the scientific engine Google Scholar (http://scholar.google.com/), and via the databases, PubMed (http://www.ncbi.nlm.nih.gov/pubmed). The following Chinese websites, http://acad.cnki.net/Kns55/brief/result.aspx?dbPrefix=CJFQ from China National Knowledge Infrastructure (CNKI), http://g.wanfangdata.com.cn/ from WanFang, and http://qikan.cqvip.com/ from WEIPU, were also searched.

## 2. Current Treatment For Radiation-Induced Brain Dysfunction

Radiation-induced neuronal apoptosis results from oxidative stress, and antioxidant treatments prevent radiation-induced brain damage [13]. Amifostine has been found to decrease the reactive oxygen species (ROS) levels [14] and suppress radiation-induced cell death in developing cerebellar granular cells [15]. Amifostine significantly attenuated recognition memory defects in adult mice exposed to low dose radiation [16] and has been widely used as a radioprotective agent.

Irradiation induces the activation of inflammatory cells and the release of inflammatory cytokines [17]. Anti-inflammation therapy has been proved to be radioprotective. Eicosapentaenoic acid is an anti-inflammation agent and could effectively protect hippocampal neurons from damage by whole body irradiation [18, 19]. Pretreatment with anti-inflammatory drugs such as indomethacin or a peroxisome proliferator-activated receptor-*α* agonist combined with fenofibrate prevented microglial activation and impairment of neurogenesis [20]. Treatment with the angiotensin converting enzyme inhibitors AT1RA L-158,809 and ramipril ameliorated radiation-induced cognitive deficits and reduced apoptosis among subgranular zone (SGZ) progenitors and inflammatory disruption within the SGZ microenvironment in the rat model [21, 22]. The administration of atorvastatin combined with ramipril appeared to synergistically ameliorate radiation-induced inhibition of neurogenesis [23]. Therefore, anti-inflammatory therapy may be a potential therapeutic approach for radiation-induced brain injury.

## 3. Traditional Chinese Medicine (TCM) with Neuroprotective Effect

Several TCM have been tested for their neuroprotective activity after brain insults (Table 1).

### 3.1. *Morinda officinalis*

Bajisin is a glycoside monomer extracted from TCM* Morinda officinalis*. It protects brain cells and has antiaging and antidepression effect. In the rat model of acute cerebral ischemic injury, Bajisin increased the activity of superoxide dismutase (SOD), glutathione peroxidase, and glucose production and reduced lipid peroxide in the brain tissue of senile mice. It had no obvious influence on the nitrogen monoxide (NO) [24]. In D-galactose and sodium nitrite-induced Alzheimer disease model, Morinda officinalis significantly increased the learning and memory ability [25]. It also had antiaging effect. Morinda officinalis significantly decreased malondialdehyde (MDA) and the apoptotic index of Purkinje fibers [26]. So far, five compounds from Morinda officinalis were isolated and their structures were identified as asrubiadin-1-methylether (I), 2-hydroxy-1-methoxyanthraquinone (II), scopoletin (III), isofraxidin (IV), and anthraquinone-2-aldehyde (V) [27]. When the forced swimming tests in mice and rats and differential-reinforcement-of-low-rate 72 second schedule (DRL72 s) in rats were used, the extracts induced significant reduction in the immobility periods in the forced swimming tests and elicited significant increases in reinforcers in DRL72 s [28]. Clinical trials suggested that Bajitian oligosaccharide capsule improved the symptoms of patients with mild or moderate depression. The efficacy was similar to fluoxetine, but it produced fewer side effects [29, 30].

### 3.2. *Lycium barbarum*

Regulates immunity, has antiaging effect, and is able to scavenge free radicals. Administration of Lycium barbarum juice significantly improved learning and memory ability and increased the activities of acetylcholinesterase (AchE) and SOD, while the contents of MDA in brains decreased obviously when compared to aging mice [31]. It increased the learning and memory ability in manganese [32] or lead [33] poisoning mice model. Lycium barbarum could also induce differentiation of bone marrow stromal cells (BMSCs) into neurons [34].

### 3.3. *Safflor*

Improved the learning and memory ability of AD rats induced by AB1-42[35]. This effect may be related to the decrease of oxidative stress and the increase of cholinergic nerve function in brain tissue [36–38].

### 3.4. *Epimedium*

Is a genus of flowering plants in the family Berberidaceae. The active component of Epimedium extracts Icariin inhibits tumor, enhances immunity, improves heart and cerebral vessels, and regulates endocrine function. Recent studies suggest that Icariin also has neuroprotective effects in the central nervous system. Icariin increased the SOD activity of brain tissue, reduced MDA and AchE activity, and therefore protected the hippocampus and improved the learning and memory ability in the rat model [39]. Icariin also promoted neurogenesis in the dentate gyrus (DG) of the hippocampus [40].

### 3.5. *Radix polygalae*

Is usually used for the treatment of human insomnia or coughing. In the rat model of Alzheimer's disease (AD), Radix polygalae could effectively improve learning and memory ability by inhibiting the activity of brain AchE, reducing MDA, free radical levels, and oxidative stress injury, and increasing SOD [41]. It significantly decreased the escape latency in hidden platform and increased the time spent in target quadrant and the number of crossing times in the spatial probe test [42]. Radix polygalae also protected neurons from the toxic effect of AB1-40 and reduced the hyperphosphorylation of tau (Ser 396) in the neurons of AD rats by activating the expression of protein phosphatase 2A (PP2A) and inhibiting the expression of protein kinase A (PKA) [43]. The main active component of Radix polygalae, Tenuigenin, promoted the differentiation of neural stem cell into nerve cells [44, 45].* Radix polygalae* improved hippocampal-dependent learning and memory and had potential antidepressant properties [46–48].

### 3.6. *Acrous tatrinouill shotts*

Is widely used in clinical practice in epilepsy, fever, phlegm syncope, faint, forgetful, stroke aphasia, tinnitus, and Alzheimer's disease. B-asarone is its main ingredient.* Acrous tatrinouill shotts* can improve the learning and memory ability of rats induced by scopolamine, which may be linked to the reduction of the expression of glial fibrillary acidic protein (GFAP) and MDA in hippocampal astrocytes [49]. Glutamate exposure to cultured rat cortical neurons induced morphological changes and lactate dehydrogenase (LDH) leakage, increased intracellular calcium concentration, and decreased cell survival rate. B-asarone could reduce intracellular calcium concentration, LDH leakage, and apoptosis ratio and therefore increase cell survival [50]. The depression was relieved effectively by B-asarone in the rat model which may be related to the improvement of the expression of Bcl-2, brain-derived neurotrophic factor (BNDF), tyrosine kinase receptor B (TrkB), and mitogen-activated protein kinases (MAPK) [51].

### 3.7. *Polygona sibiricum*

Has antiaging, lowering increasing coronary blood flow, and anticancer effect. Polygona sibiricum improved the learning and memory ability in mice. Alcohol extract from the rhizome of Polygonatum sibiricum improved acquisition of impairment of memory induced by scopolamine (SCO) in mice. It also extended the survival time of mice subjected to cerebral ischemia by the occlusion of the bilateral carotid arteries and that PS ethanolic extract 2.0 mg/mL and 10.0 mg/mL inhibited MDA formation in the rat brain tissue [52]. Polygona sibiricum also reduced the deposition of A*β* in the hippocampus of AD rats [53].

### 3.8. *Cynomorium songaricum Rupr*

Has the role of tonifying kidney yang, enhancing aphrodisiac action, and laxative effect. It markedly improved the learning and memory ability in the rat AD model by reducing oxidative stress in the brain tissue and promoting the formation of synapses [54–56].

### 3.9. *Alpinia oxyphylla miq*

Is a dried ripe fruit of the perennial plant of the ginger family. It significantly improved the learning and memory ability in mice induced by scopolamine [57]. The Alpinia oxyphylla Miq could prevent the injury of hippocampal CA3 neurons in the restrained stress rat model [58].

### 3.10. *Broomrape*

Has different biological activities, such as immune regulation, memory enhancement, antioxidative, antiaging, and radiation protection. A significant improvement of memory was observed in mice with learning disabilities induced by hydrocortisone [59, 60]. Broomrape protected the brain cells by scavenging oxygen free radicals and reducing lipid peroxidation damage to brain tissue [61, 62]. It improved the learning and memory of AD mice by decreasing the content of MDA, increasing the activity of SOD, GSH-Px, and decreasing the activity of AChE, the apoptosis rate of brain cells, and the accumulation of calcium in brain tissue [63–65]. In AD patients, Broomrape improved the cognitive and self-care ability and delayed the progress of dementia [66]. The total glycosides of Cistanche deserticola extract could significantly improve the behavioral characteristics in the mouse Parkinson's disease model induced by 1-Methyl-4-phenyl-1,2,3,6-tetrahydropyridine (MPTP) and increase the content of dopamine in striatum and the expression of tyrosine hydroxylase in the substantia nigra [67].

## 4. Traditional Chinese Medicine with Radioprotective Effects

### 4.1. Astragalus membranaceus

Hydroponically grown root extracts from Astragalus membranaceus significantly reduced UVA-induced DNA damage in cultured human lung and skin fibroblasts [68]. In the brain of rats with acute encephalopathy caused by ^60^Co irradiation, the intraperitoneal injection of Astragalus parenteral solution decreased the nitric oxide and ameliorated the cognitive ability, suggesting that astragale may protect radiation-induced brain injury [69, 70]. The acute and chronic electromagnetic field (EMF) irradiation could initiate neurologic damage in hippocampus. Chinese medicine diet (CMD) which comprised ferulic acid, gimenoside, astragalus polysaccharide, an ingredient of Astragalus, and rhodiola sachaliens has protective effect on the impaired learning and memory, the neuronal apoptosis, and the peroxidation damage induced by electromagnetic field irradiation. CMD intervention played a significant protective role in antagonizing neurological damage in the later stage of acute irradiation and chronic irradiation [71, 72]. Astragalus also significantly protected neuronal apoptosis induced by radiation injury at a single-dose X-ray exposure of 30 Gy [73]. While radiotherapy prolongs the survival time of patients with head and neck tumor, the side effects such as radiation optic neuropathy may lead to irreversible visual loss which seriously affects the quality of life of patients. Recent studies suggested that Astragalus membranaceus significantly improved the visual acuity of irradiated rats or patients with nasopharyngeal carcinoma after radiotherapy [74, 75].

### 4.2. Salvia miltiorrhiza

The bioactive constituents of Salvia miltiorrhiza, i.e., tanshinones and depsides, protect against *β*-amyloid-induced toxicity by the anti-inflammatory mechanisms. The two constituents enhance the antiapoptotic B-cell leukemia protein-2 family members, decrease the translocation of cytochrome* c*, and have an activity on vascular endothelial growth factor. In addition, depsides decrease caspase-3, intracellular Ca(2+), and reactive oxygen species while tanshinones enhance the activities of superoxide dismutase and glutathione peroxidase [76]. In the mouse whole brain irradiation model, Salvia miltiorrhiza prevented the high dose radiation-induced brain structural and functional changes and improved the quality of life by ameliorating the primary events [77]. Microwave irradiation induced a significant decrease of ATPase activity and a remarkable increase of Na^+^, Ca^2+^ contents in the hippocampus. However, Salvia miltiorrhiza could significantly lower the inhibition of ATPase activity and the increase of Na^+^, Ca^2+^ in the hippocampus. The neuronal damage was also ameliorated substantially [78]. The behavioral test indicated that Salvia miltiorrhiza could improve the learning and memory ability of rats [79]. This was confirmed by another study showing that Salvia miltiorrhiza improved the ionizing radiation-induced cognition impairment by reducing lipid peroxide (LPO) and intercellular cell adhesion molecule-1 (ICAM-1) expression in the mouse model [80]. The brain radioprotective effect of Salvia miltiorrhiza was also confirmed in clinical studies showing that radiotherapy combined with administration of Salvia miltiorrhiza significantly reduced radiation-induced brain injury [81–83].

### 4.3. Ligusticum chuanxiong Hort

Ligusticum chuanxiong Hort and its bioactive ingredient, tetramethylpyrazine (TMP), have been used to treat cardiovascular diseases and to relieve various neurological symptoms. TMP effectively protected neuronal apoptosis, which was associated with the inhibition of oxidative stress and a change in the levels of apoptosis-related proteins, Bcl-2 and Bax. Furthermore, TMP reduced the expression of proinflammatory cytokines such as TNF-*α* and IL-8, which likely contributes to its cytoprotective effects [84]. Clinical study has shown that TMP significantly improves the symptoms of patients with radiotherapy-induced encephalopathy [85] and optic neuropathy [86].

### 4.4. Broomrape

Citanche glycoside is an active component of Broomrape. It facilitated the repairing process of radiation-induced damage to the biological membranes and cells of sensitive organs in the mice [87].

### 4.5. Horse Chestnut P.E.

*β*-aescine sodium is an ingredient from Horse Chestnut P.E. Cerebral edema is a radiation injury at acute stages after the exposure. Early aggressive treatment of cerebral edema could relieve the symptoms of intracranial hypertension, delay or block the development of the disease, and prevent the occurrence of cerebral hernia. Clinical data indicated that patients with radiation-induced brain edema could be effectively controlled by *β*-aescine sodium or mannitol and dexamethasone [88].

### 4.6. Radix Hedysari

In *γ*-ray irradiated rat model, administration of Radix hedysari significantly increased SOD, but decreased MDA activity in the brain tissue. It suggests that Radix hedysari may serve as an antioxidant drug and be helpful for the recovery of radiation-induced brain damage [89, 90]. Radix hedysari capsule increased the wet weight of liver, spleen, brain, and testicular tissue of irradiated mice, suggesting that it has radioprotective effect [91].

### 4.7. Safflower

^12^C^6+^ irradiation induces cognitive dysfunction and impairment of the blood brain barrier, significantly decreased SOD, and increased MDA activity in the brain tissue. Hydroxysafflor yellow A, an ingredient of safflower, dose dependently improved cognitive dysfunction, protected the blood-brain barrier, increased SOD, and decreased MDA activity. It suggests that hydroxysafflor yellow A may have a radioprotective effect on radiation-induced brain injury [92].

### 4.8. Arnebiae Radix

Shikonin, a bioactive ingredient of Arnebiae Radix, improved ^12^C^6+^ ion beam-induced brain injury by its modulating effects on the oxidative stress [93].

### 4.9. Ginkgo

Ginkgo flavonoid, an extract from Ginkgo, prevented age-related spatial memory deficits in both animal study [94] and clinical trial [95]. After high dose irradiation, Ginkgo flavonoid could prevent the radiation-induced hippocampal injury [96].

### 4.10. Ginseng

Panaxoside Rgl, a bioactive ingredient of Ginseng, reduced neuron apoptosis by controlling Cdk5 and played a protective role in radiation-induced hippocampal damage [97].

### 4.11. Kang-fu-ling

Kang-fu-ling (KFL) is a polybotanical dietary supplement with antioxidant properties. KFL reversed high power microwave-induced memory loss and the histopathological changes in hippocampus of rats. In addition, KFL displayed a protective effect against HPM-induced oxidative stress and activated the nuclear factor-E2-related factor 2 (Nrf2) and its target genes in the hippocampus of rats. The Nrf2-antioxidant response element (ARE) signaling pathway may be involved in the neuroprotective effects of KFL against HPM-induced oxidative stress. The dietary supplement KFL may therefore be a promising natural radioprotector [98].

### 4.12. Shenqi

Shenqi Fuzheng Injection (SFI) was extracted from a number of medicinal herbs, such as Radix Codonopsis (root of Codonopsis pilosula) and Radix Astragali (root of Astragalus), and was approved by the State Food and Drug Administration of China in 1999. Administration of SFI effectively attenuates irradiation-induced brain injury via inhibition of the NF-*κ*B signaling pathway and microglial activation [99].

### 4.13. 978-1

A TCM of destagnation and renal invigoration (978-1) was effective to prevent or treat the damage of learning and memory ability caused by irradiation in mice. It was able to prevent or treat radiation-induced subventricular cell damage by downregulation of p53 and C-jun expression and inhibition of apoptosis [100, 101].

## 5. The Molecular Mechanisms of Neuroprotective and Radioprotective Effect of TCM

It has been well documented that radiation induces brain oxidative stress, microglial activation, acute and chronic inflammatory responses, apoptosis, autophagy, abnormal angiogenesis and neurogenesis, redistribution or imbalanced neurotransmitter and receptor systems, and downregulation of neural growth factors [102]. TCM may play roles in antioxidative stress and antiapoptosis. It could promote hippocampal neurogenesis, improve microcirculation, inhibit microglial activation and inflammation, and reduce TAU production (Table 2).

The roles of antioxidative stress and antiapoptosis of TCM have been well accepted. Morinda officinalis increases the activity of superoxide dismutase (SOD) and glutathione peroxidase (GSH-Px) in the brain tissue of rats with acute cerebral ischemia and aging and reduce the content of lipid peroxide (LPO) [24, 26]. In the Lycium barbarum-treated aging mice induced by D-galactose, the animal learning, memory, and brain AchE and SOD increased significantly, while brain MDA decreased obviously. Similar changes were also found in Lead poisoning mice [31, 33]. Safflor increased SOD and GSH-Px, but decreased MDA in the cortical tissue of AD rats [35–38, 92]. Epimedium improved animal learning and memory ability by increasing brain SOD, decreasing MDA and Ach E, and reducing the damage of hippocampal neurons by D-gal AlCl_3_ treatment [39]. In the AD model induced by the hippocampal injection of amyloid-*β*25~35, the brain SOD were decreased, but AchE and MDA were increased obviously leading to learning and memory impairment. Radix polygalae treatment increased SOD, reduced AchE and MDA, and improved learning and memory ability [41]. Similarly, Acrous tatrinouill shots [49, 51, 103], Polygona sibiricum [52], and Cynomorium songaricum Rupr [54–56, 104, 105] also increased the brain SOD, reduced MDA, and improved the impairment of learning and memory, whereas Alpinia oxyphylla miq. fruit, Broomrape, and Astragalus membranaceus are antioxidative and antiapoptotic [57–75, 87], and Salvia miltiorrhiza decreased the brain lipid peroxidase in AD rats [76, 80, 81]. Ligusticum chuanxiong Hort inhibited free radicals in hypoxia and reduced neuronal apoptosis [85]. In ^60^Co-*γ* irradiated animals, Radix Hedysari treatment increased the brain superoxide dismutase activity but reduced maleic dialdehyde [89, 90]. Other antioxidative stress and antiapoptosis TCM include Arnebiae Radix, Ginkgo, Ginseng, Kang-fu-ling, Shenqi, and Renal invigoration (978-1) [93–101].

TCM may also promote neurogenesis to prevent radiation-induced cognitive impairment. Morinda officinalis and Lycium barbarum promoted neurogenesis in the subgranular zone of the dentate gyrus [29, 32, 34]. Morinda officinalis also increased the number of dendrites and their branches of the hippocampal neurons [29], whereas Lycium barbarum had inductive effect on differentiation of bone marrow stromal cells (BMSCs) into neurons [32, 34]. Epimedium significantly reduced senile plaques in the hippocampus and increased the number of BrdU+ cells in the dentate gyrus. Our previous study showed that epimedium extract prevented the loss of proliferation cells, newly generated neurons, and interneurons in the hilus, in particular, the subgranular zone of the dentate gyrus [40, 106]. In vitro study showed that adding tenuigenin to the neural stem cell medium increased the number of newly formed neurospheres and promoted the differentiation of the hippocampal neural stem cells (NSCs) into neurons [44–46, 48].

TCM improved radiation-induced inflammation and microcirculation changes. TMP reduced the expression of proinflammatory cytokines such as TNF-*α* and IL-8, which may also contribute to its cytoprotective effects [84]. Horse Chestnut P.E. improved microcirculation and stabilizing vascular endothelial cells and has been used to treat radiation-induced brain edema [88]. In rats with AB25-35 induced Alzheimer's disease, Epimedium was used to improve spatial learning and memory by inhibiting TNF-a, IL-6, and caspase-3 expression [107]. Radix polygalae reduced the brain tau level, the phosphorylation of tau protein, and the expression of PKA, but increased the expression of PP2A in amyloid *β* peptide 1-40 (A*β*_1-40_)-injected mice [43]. Salvia miltiorrhiza inhibited the radiation-induced senile plaques and neurofibrillary tangles in the mouse brain [77], whereas Shengqifuzheng effectively attenuated irradiation-induced brain injury by inhibiting NF-*κ*B signaling pathway and microglial activation [99].

**Figure 1:**
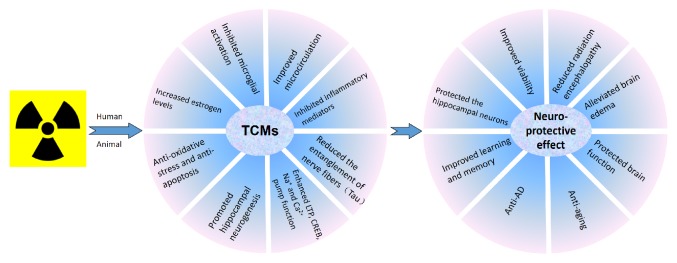
Traditional Chinese Medicine (TCM) reduces radiation-induced microglial activation and production of inflammatory cytokines and chemokines to inhibit brain inflammation. TCMs increase the superoxide dismutase activity and decrease malondialdehyde and nitric oxide production to reduce oxidative stress. Upregulation of Bcl-2, downregulation of Bax-2, downregulation of p53 and c-jun expression, and increased ratio of Bcl-2/Bax by TCMs prevent radiation-induced apoptosis. TCMs may also increase brain-derived neurotrophic factor (BNDF), tyrosine kinase receptor B (TrkB), and mitogen-activated protein kinases (MAPK) to promote neurogenesis and improve radiation-induced impairment of cognition. These drugs also enhance LTP, CREB, Na^+^ and Ca^2+^ pump function, increase estrogen levels, and reduce the entanglement of nerve fibers (Tau) to improve radiation-induced neurocognitive impairment.

TCM may increase estrogen level and change the Ca^2+^ Na^+^ function to protect brain injury. Acrous tatrinouill shots could reduce intracellular calcium concentration [50]. The oral administration of icariin increased serum estradiol(E_2) level and improved the learning and memory ability in AD rats [108]. Salvia miltiorrhiza increased ATPase activity and reduced Na+, Ca2+ in the hippocampus (CA1 area) and improved microwave radiation-induced brain damage [78] (refer to Figure 1).

## 6. Limitation of TCM as Radio-Neuro-Protectants

While it seems promising to use TCM as potential radio-neuro-protectants, it should be emphasized that the ingredients of TCM are very complex and have not been fully identified and purified. Improper processing, dispensing, compatibility, excessive dosage, and individual differences may significantly affect clinical use of TCM [109]. As individual reponse to a same TCM may be different, it may compromise the usage of TCM for treatment of victims with massive radiation exposure. TCM Formulae with two or more herbs often produce better curative efficacies and fewer side effects than a single herb, but improper use may produce more harm than benefit [110]. Disbelief of TCM may also limit its use worldwidely.

## 7. Conclusions and Future Research Directions

Extensive publications on neuroprotective and radioprotective effect of TCM suggest that TCM may be effective in prevention of radiation-induced glial cell activation and proliferation, neuroinflammation, oxidative stress, apoptosis, and neurodegeneration. TCM may also promote brain neurogenesis and improve radiation-induced impairment of cognition. However, different doses and combination of TCM, animal species, strains, ages, and sexes were used in different research laboratories in previous studies. The variations in radiation sources, doses/dose rates, and irradiation patterns (acute or fractionated) made it difficult to evaluate if the positive effect of TCM in one animal model or laboratory could be applied to other models or laboratories. The neuroprotective or radioprotective effect of TCM administered before irradiation may not be observed when TCM are injected after irradiation. The routes of TCM administration, i.e., oral or intraperitoneal injection, may also compromise the data translation. Furthermore, the purity of the components and composition of the compounds of TCM were not clearly mentioned in most of the previous studies which may limit its clinical use.

TCM with radioprotective effect on the brain are far less investigated than on other organs. Further extensive studies in the following areas may still be needed: (1) Radiosensitivity varies significantly among different strains of animals. The effect of TCM may have to be tested in the same strain of animals for comparison in order to make solid conclusions. Animal age and sex should be chosen carefully as TCM effect may be age- and sex-dependent. (2) When animals are exposed to the same dose of radiation, radiation dose rate may affect radiosensitivity. Therefore, the same dose rate of radiation exposure should be used to compare TCM effect. The radiation source and component of radiation may also affect TCM effect. (3) The mechanism of high dose/dose rate radiation-induced brain damage may be different from low dose/dose rate radiation-induced injury. Comparative study of radioprotective effect of TCM in animals exposed to high and low doses of radiation exposures, in particular, the latter, may shed light on further understanding the mechanisms of the two patterns of radiation-induced brain damage.

## Figures and Tables

**Table 1:** Neuroprotective effect of Traditional Chinese Medicine (TCM) after brain insults.

**No.**	**Herbs and plant extract**	**Main acting constituents**	**Test system**	**Brain insults**	**Effect**	**Mechanism**	**References**
**1**	**Morinda officinalis**	Rubiadin, Rubiadin-1-methylether, etc.	Rat, mouse, human	Alzheimer's disease (AD), memory disorder	Improve learning and memory, Anti-AD, protect brain function, anti-depression, anti-aging, improve language function	Anti-oxidative stress, anti-apoptosis, enhanced LTP function in hippocampal synapse, improving glucose metabolism	[24–30]

**2**	**Lycium barbarum**	Lycium barbarum polysaccharide, Betane, etc.	Rat, mouse	Memory disorder resulted by lead expose or manganese poisoning	Improve learning and memory, protect brain function	Anti-oxidative stress, anti-apoptosis, promoting hippocampal neurogenesis	[31–34]

**3**	**Safflor**	Carthamin, Safflow yellow, etc.	Rat, mouse	Memory disorder	Improve learning and memory	Anti-oxidative stress, anti-apoptosis,	[35–38]

**4**	**Epimedium**	Icariin, Icariside, etc.	Rat, mouse	Dementia	Improve learning and memory, Anti-AD, protect brain function, anti-aging	Anti-oxidative stress, anti-apoptosis, reducing the entanglement of nerve fibers (Tau), promoting hippocampal neurogenesis, inhibiting inflammatory mediators, increase estrogen levels, increase the activity of sodium pump and calcium pump	[39, 40]

**5**	**Radix polygalae**	Onjisaponin, Ketone, etc.	Rat, mouse	AD	Improve learning and memory, Anti-AD, protect brain function	Anti-oxidative stress, anti-apoptosis, enhancing LTP function in hippocampal synapse, increasing the expression of CREB in the hippocampus, inhibiting inflammatory mediators, improve the entanglement of nerve fibers(Tau) promoting hippocampal neurogenesis	[41–48]

**6**	**Acrous tatrinouill shotts**	*β*-Asarone, *α*-Asarone, etc.	Rat, mouse	Memory impairment by scopolamine; cortical neuron damage; depression	Improve learning and memory, Anti-AD, protect brain function, anti-depression	Anti-oxidative stress, anti-apoptosis, increasing the expression of CREB in the hippocampus	[49–51]

**7**	**Polygona sibiricum**	Polygonatum Polysaccharide, Street soap shake, etc.	Rat, mouse	Memory impairment by scopolamine	Improve learning and memory, Anti- AD, protect brain function, anti-depression	Anti-oxidative stress, anti-apoptosis, improving cerebral ischemia	[52, 53]

**8**	**Cynomorium songaricum Rupr**	Anthocyanin, Triterpenoid saponins, etc.	Rat, mouse	AD	Improve learning and memory, Anti-AD, protect brain function, anti-aging	Anti-oxidative stress	[54–56]

**9**	**Alpinia oxyphylla miq. Fruit**	Sesquiterpene, Monoterpene, etc.	Rat, mouse	Scopolamine-induced dementia	Improve learning and memory, protect brain function, anti-aging	Anti-oxidative stress, anti-apoptosis	[57, 58]

**10**	**Broomrape**	Ergosterin, Cistanche glycoside, etc.	Mouse, human	AD; Memory impairment; cerebral ischemia	Improve learning and memory, protect brain function	Anti-oxidative stress, anti-apoptosis	[59–67]

**Table 2:** Neuroprotection effect of TCM after radiation exposure.

**No.**	**Herbs and plant extract**	**Main acting constituents**	**Test system**	**Brain insults (radiation source, dose, dose rate)**	**Effect**	**Mechanism**	**References**
**1**	**Astragalus membranaceus**	Astragalus polysaccharides, Total saponins of Astragalus, etc.	Rat, mouse, Human, cell	^60^Co, 4.5Gy/min, 160-170s, one time, single-dose X-rays exposure of 30 Gy	Improve learning and memory, increase SOD activity, decrease MDA	Scavenge oxygen free radicals, reduce nitric oxide production	[68–75]

**2**	**Salvia miltiorrhiza**	Tanshinone, Cryptotanshinone, etc.	Mouse, Rat, Human	Varian-600, X-rays, 22Gy	Improve learning and memory	Reduce lipid peroxide in brain tissue and inhibit the adhesion of endothelial cells factor 1 expression	[76–83]

**3**	**Ligusticum chuanxiong Hort**	Chuanxiongzine, Ligustilide, etc.	Human	Patients had a history of radiotherapy for head and neck cancer	Reduce radiation encephalopathy	Improve microcirculation, expand blood vessels, inhibit the generation of oxygen free radicals	[84–86]

**4**	**Broomrape**	Ergosterin, Cistanche glycoside, etc.	Mouse	5 Gy 60Co-*γ*	Improve viability	Scavenge oxygen free radicals, Strengthen immunity	[87]

**5**	**Horse Chestnut P.E**	Aescine, etc.	Human	Patients had a history of radiotherapy for head cancer	Prevent brain edema	Stabilize endothelial cells,	[88]

**6**	**Radix Hedysari**	Hedysarum polysaccharide, etc.	Rat, Mouse	2 Gy 60Co-*γ*	Increase SOD activity, decrease MDA	Reduce oxidative stress	[89–91]

**7**	**safflower**	Carthamin, Safflow yellow, etc.	Mouse	4 Gy ^12^C^6+^	Increase SOD activity, decrease MDA	Reduce oxidative stress	[92]

**8**	**Arnebiae Radix**	Shikonin, Acetylshikonin, etc.	Mouse	^12^C^6+^ ion beam, dose rate of approximately 0.5 Gy/min	Improve the spatial memory deficits	Reduce oxidative stress	[93]

**9**	**Ginkgo**	Flavonoids, ginkgolides, etc.	Rat	12MEV, 20 Gy	Inhibition of brain cell edema	Scavenge oxygen free radicals, apoptosis inhibition	[94–96]

**10**	**Ginseng**	Ginsenoside, etc.	Rat	30 Gy	Protect the hippocampal neurons	Inhibit apoptosis	[97]

**11**	**Kang-fu-ling**	Total glucoside of Astragalus, total glucoside of Radix Paeoniae Rubra and Tanshinone	Rat	HPM at 30 mW cm ^2^ for 15 min	Improve spatial memory	Modulate ROS formation and antioxidant enzymes.	[98]

**12**	**Shenqi**	Codonopsis polysaccharides, Astragalus polysaccharides, etc.	Mouse	A single dose of cranial radiotherapy (CRT) with 20Gy	Improve the physical status, survival, and spatial learning, attenuate all the CRT-induced changes in the brain tissues.	Inhibit NF-*κ*B signaling pathway and microglial activation	[99]

**13**	**Renal invigoration (978-1)**	Icariin, Lignin, etc.	Mouse	A single dose of 20 Gy	Prevent impairment of learning and memory	Inhibit apoptosis	[100, 101]
